# Quantitative spinal cord MRI and sexual dysfunction in multiple sclerosis

**DOI:** 10.1177/20552173221132170

**Published:** 2022-10-20

**Authors:** Estelle Seyman, David Kim, Aditya Bharatha, Courtney Casserly, Kristen Krysko, Roy-Hewitson Chantal, Paula Alcaide-Leon, Suradech Suthiphosuwan, Jiwon Oh

**Affiliations:** Division of Neurology, Department of Medicine, 10071 St Michael's Hospital, 7938 University of Toronto, Toronto, Ontario, Canada; Division of Neurology, 26738 Tel Aviv Sourasky Medical Center, Tel-Aviv University, Tel-Aviv, Israel; Department of Clinical Neurological Sciences, London Health Sciences Centre, Western University, London, Ontario, Canada; Division of Neuroradiology, Department of Medical Imaging, St. Michael's Hospital, 7938 University of Toronto, London, Ontario, Canada; Department of Clinical Neurological Sciences, London Health Sciences Centre, Western University, London, Ontario, Canada; Division of Neurology, Department of Medicine, 10071 St Michael's Hospital, 7938 University of Toronto, Toronto, Ontario, Canada; Division of Neuroradiology, Department of Medical Imaging, St. Michael's Hospital, 7938 University of Toronto, London, Ontario, Canada; Division of Neurology, Department of Medicine, 10071 St Michael's Hospital, 7938 University of Toronto, Toronto, Ontario, Canada; Department of Neurology, 1466 Johns Hopkins University, Baltimore, Maryland, USA

**Keywords:** multiple sclerosis, sexual dysfunction, quality of life, spinal cord, quantitative MRI

## Abstract

**Background:**

Sexual dysfunction (SD) is frequently reported in multiple sclerosis (MS) and is likely related to MS-related damage to the spinal cord (SC).

**Objective:**

To assess associations between SD and quantitative MRI measures in people with MS (pwMS).

**Methods:**

This pilot study included 17 pwMS with SD who completed questionnaires assessing SD, mood, and fatigue. All participants underwent brain, cervical, and thoracic SC-MRI at 3T. Quantitative brain and SC-MRI measures, including brain/SC atrophy, SC lesion count, diffusion-tensor imaging (DTI) indices (fractional anisotropy [FA], mean, perpendicular, parallel diffusivity [MD, λ_⊥_, λ_||_]) and magnetization-transfer ratio (MTR) were obtained. Associations between quantitative MRI measures and SD were assessed while controlling for the extent of mood and fatigue symptomatology.

**Results:**

Subjects were a mean age of 46.9 years and 29% female. All subjects had self-reported SD (MSISQ-19 = 40.7, SQoL: 55.9) and 65% had a concurrent psychiatric diagnosis. When correlations between SD severity were assessed with individual brain and SC-MRI measures while controlling for psychiatric symptomatology, no associations were found. The only variables showing independent associations with SD were anxiety (*p* = 0.03), depression (*p* = 0.05), and fatigue (*p* = 0.04).

**Conclusion:**

We found no correlations between quantitative MRI measures in the brain and SC and severity of SD in pwMS, but psychiatric symptomatology and fatigue severity demonstrated relationships with SD. The multifactorial nature of SD in pwMS mandates a multidisciplinary approach.

## Introduction

Multiple sclerosis (MS) is a chronic, inflammatory, demyelinating disorder of the central nervous system that can result in a variety of symptoms and progressive neurological disability over time. MS is one of the most common chronic neurological disorders among young adults, and sexual dysfunction (SD) is frequently reported, with estimates ranging between 31% and 92% in both women and men with MS.^1–5^ Sexual function is an important component of health-related quality of life (HRQoL),^1,2,6^ and SD has been shown to have detrimental effects on the mental and physical aspects of QoL in people with MS (pwMS).^2,5,7,8^ Individuals with SD report poor outcomes in all major dimensions of well-being, including health, sense of fulfillment, and personal relationships.^7,8^

MS-related SD can be divided into three main categories: (1) primary: due to MS-related neurological deficits that directly affect the sexual response, (2) secondary: due to MS-related physical impairments and symptoms that indirectly affect sexual response (i.e., spasticity, fatigue, bladder dysfunction, and cognitive dysfunction), and (3) tertiary: due to the psychological, social, and cultural issues related to having a chronic disease and perceived disability.^9^ Despite its high prevalence in MS, SD is infrequently discussed by physicians and patients^10^ with one study reporting only 2.2% of patients ever recalling having discussed sexual concerns with their physician.^11^

Mood symptoms and fatigue are commonly observed in pwMS and negatively impact HRQoL.^12,13^ Given that mood disorders and fatigue are linked to secondary and tertiary SD in MS,^14^ they should be taken into account when assessing SD in the pwMS.

The spinal cord (SC) is a compact structure with a functional-anatomic organization that mediates specific neurological functions, including sexual function. Although SD in MS is multifactorial, it is at least in part mediated by damage to the SC and its autonomic connections.^3,15,16^ Thus, the SC is an ideal structure to better understand structure–function relationships and anatomic correlates of SD in pwMS.

Historically, technical difficulties and limited clinical-radiological correlations have made the SC challenging to study using conventional, lesion-based MRI measures.^17,18^ Advanced, quantitative MRI techniques in the SC, including diffusion-tensor imaging (DTI) and magnetization-transfer imaging (MTI) have demonstrated increased sensitivity to tissue microstructural properties in comparison to conventional MRI techniques and have the potential to provide clinically relevant information on tissue integrity beyond that which can be obtained from conventional MRI measures alone.^19,20^ To date, only one study (*n* = 30) has examined the association between a quantitative SC-MRI measure (spinal cord atrophy) and SD in pwMS and failed to find a correlation between SD and SC cross-sectional area (CSA).^16^ Other MRI studies in small cohorts of pwMS with SD failed to find an association with the location and number of lesions in the SC,^21^ though inconsistent associations were found with different brain MRI measures including lesion load, location, and brain atrophy measures.^16,21–25^ To our knowledge, there are no published studies assessing advanced SC-MRI measures such as DTI and MTI in pwMS with SD.

The objective of this pilot study was to evaluate advanced, quantitative brain and SC-MRI measures in pwMS with SD and to assess for correlations between SD severity and these measures.

## Methods

This study was approved by the Institutional Review Board of St Michael's Hospital. All participants provided written informed consent. Seventeen pwMS, meeting the 2017 McDonald diagnostic criteria^26^ with self-reported SD, were prospectively recruited between January 2015 and July 2017. Participants were referred to the study by their treating neurologist if they endorsed symptoms of SD and were interested in participating in research, or by self-referral as there were advertisements of the study posted in the clinic. Exclusion criteria included those unable to undergo an MRI or pregnant at the time of recruitment were excluded from the study. All patients who were referred for the study met inclusion and exclusion criteria.

*Clinical assessment.* Each participant attended a single study visit and underwent an MRI of the brain, cervical, and thoracic SC at 3T. Clinical information was collected and a neurological examination was performed by a neurostatus-experienced examiner to obtain the Expanded Disability Status Scale (EDSS) score.^27^ Participants were asked to complete multiple questionnaires validated for use in pwMS quantifying sexual QoL; (Multiple Sclerosis Intimacy and Sexuality Questionnaire-19 [MSISQ-19], Sexual Quality of Life Questionnaire [SQoL-M for men; SQoL-F for women], International Index of Erectile Function [IIEF] for men, Female Sexual Function Index [FSFI] for women). Additionally, participants completed questionnaires quantifying mood (Beck Depression Inventory-II [BDI-II], Hospital Anxiety and Depression Scale [HADS]) and fatigue (Fatigue Impact Scale [FIS]). Further details on questionnaires and their validation in pwMS are outlined in Supplemental Table 1.

**Table 1.** Clinical and MRI characteristics of study population.

Clinical characteristics	Study patients (*n* = 17)
Age, mean (SD), years	47 (10.3)
Female sex, *n* (%)	5 (29)
Higher education, *n* (%)	8 (47)
Retired/unemployed, *n* (%)	7 (41)
Psychiatric comorbidities*, *n* (%)
	11 (64.7) Depression = 7 (63.5) Anxiety = 5 (45.5) Substance abuse = 2 (18) Paranoid psychosis = 1 (9)
Cardiovascular comorbidities, *n* (%)	4 (23.5)
Disease subtype, *n* (%)	RRMS 10 (58.8) SPMS 4 (23.5) PPMS 3 (17.7)
Disease duration, mean (SD), years	15.05 (7.83)
Spinal cord presentation, *n* (%)	11 (61)
EDSS, mean (SD)	3.0 (1.5)
Annual relapse rate, mean (SD)	0.383(0.254)
DMT, *n* (%)	No DMT, 3 (17%) On DMT, 14 (83%)
Advanced brain and spinal cord MRI measures	
Automated SC cross-sectional area, mean (SD), mm^2^	73.58 (7.09)
Cervical SC lesion count, mean (SD)	4.93 (2.99)
Thoracic SC lesion count, mean (SD)	4.64(2.20)
Number of patients with longitudinal SC lesion, *n* (%)	2 (11)
FA, mean (SD)	0.63 (0.06)
MD, mean (SD), μm^2^/ms	0.0012 (0.0001)
λ_||_, mean (SD), μm^2^/ms	0.002 (0.0002)
λ_⊥_, mean (SD), μm^2^/ms	0.0007 (0.0003)
MTR, mean (SD)	0.305 (0.034)
Brain parenchymal fraction, mean (SD)	0.805 (0.023)
Brain lesion volume, mean (SD), cc	8.54 (7.85)

SD: standard deviation; MS: multiple sclerosis; RRMS: relapsing remitting MS; SPMA: secondary progressive MS; PPMS: primary progressive MS; EDSS: expanded disability status scale; DMT: disease-modifying treatment; SC: spinal cord; FA: fractional anisotropy; MD: mean diffusivity; λ_||_: parallel diffusivity; λ_⊥_: perpendicular diffusivity; MTR: magnetization-transfer ratio.

*Two individuals had two psychiatric diagnoses, and one individual had three psychiatric diagnoses.

### MRI image acquisition protocol

MRIs of the brain, cervical, and thoracic SC were performed on a 3T MRI scanner (Siemens Skyra, Erlangen, Germany) using a 20-channel head-neck coil and a 16-channel spine-array coil.

*Brain MRI*. Brain sequences: T1-weighted magnetization-prepared rapid acquisition gradient echo (T1-MPRAGE): TR/TE/Flip angle = 1900 ms/2.52 ms/9°, parallel acceleration technique mode = GRAPPA, acceleration factor = 2, slice thickness = 1 mm, field of view (FOV) = 250 mm, in-plane resolution = 1 × 1 mm^2^, and number of slices = 176 and T2-weighted fluid-attenuated inversion recovery (FLAIR): TR/TE = 4800 ms/353 ms, TI = 1800 ms, parallel acceleration technique mode = GRAPPA, acceleration factor = 2, slice thickness = 1 mm, FOV = 256 mm, in-plane resolution = 1 × 1 mm^2^, number of slices = 176.

*Spinal cord MRI*. Sagittal 2D T1-weighted phase-sensitive inversion recovery (PSIR) of the cervical spine^28^: FOV = 220 mm; in-plane resolution = 0.7 × 0.7 mm; slice thickness = 3 mm; TR/TE = 2400/9.4 ms; TI = 400 ms; averages = 2; parallel acceleration technique mode = GRAPPA; and acceleration factor = 2.

Sagittal 2D T1-Short-TI-Inversion Recovery (STIR) of the thoracic spine: FOV = 220 mm; in-plane resolution = 0.48 × 0.48 mm; slice thickness = 3 mm; TR/TE = 4000/50 ms; TI = 200 ms; averages = 1; parallel acceleration technique mode = GRAPPA; and acceleration factor = 2.

*MTI data*. MT-weighted images (MT_on_): 3D T2*-weighted, gradient-echo sequence with an MT prepulse (1.5-kHz off-resonance sinc-gauss-shaped radiofrequency saturation pulse), flip angle/TR/TE = 9°/47 ms/11.2 ms, which yielded 3-mm axial slices (20 contiguous) spanning C3-C4 with FOV = 224 mm, and a nominal in-plane resolution of 0.6 × 0.6 mm^2^. MT_off_ images used the same parameters but excluded the MT prepulse.

*DTI data*. Cardiac-gated, axial fat-suppressed, high-resolution diffusion-weighted imaging with readout-segmented echo-planar imaging, parallel imaging, and two-dimensional navigator-based reacquisition (RESOLVE) was obtained across C3–C4 in 12 noncoplanar gradient directions. Flip angle/TR/TE = 180°/220 ms/53 ms, *b* = 500 s/mm^2^, trigger delay = 0 ms, acquisition window = 420 ms, parallel acceleration technique mode = GRAPPA, acceleration factor = 2, slice thickness = 3 mm, FOV = 150 mm, in-plane resolution = 1.5 × 1.5 mm^2^, and slice number = 20.

### MRI image processing and analysis

*Brain image analysis*. All acquired images were de-identified. Automatic brain lesion segmentation was performed using the Lesion Segmentation Toolbox^29^ for SPM8 (Department of Imaging Neuroscience, London, UK) on MATLAB software. The optimal initial threshold for segmentation was determined by comparing a reference manual segmentation with automatic segmentations created on FLAIR sequences, with different thresholds. Dice coefficients for different thresholds were calculated, and a threshold of 0.25 was selected. Lesion maps were used to fill the segmented lesions in the T1-MPRAGE image with estimated healthy white matter (WM) tissue.^30^ Gray matter (GM), WM, and cerebrospinal fluid (CSF) volumes were obtained using the voxel-based morphometry 8 (VBM8) toolbox^31^ for SPM8. Lesion, GM, WM, and CSF maps were reviewed for quality control by an experienced reader (ES). Brain parenchymal fraction (BPF), a measure of whole-brain atrophy, was calculated by dividing brain parenchymal volume (GM + WM) by total intracranial volume (GM + WM + CSF). Two subjects were excluded from the brain volume analyses due to missing sequences in the study scan and technical inadequacies.

**Figure 1.**
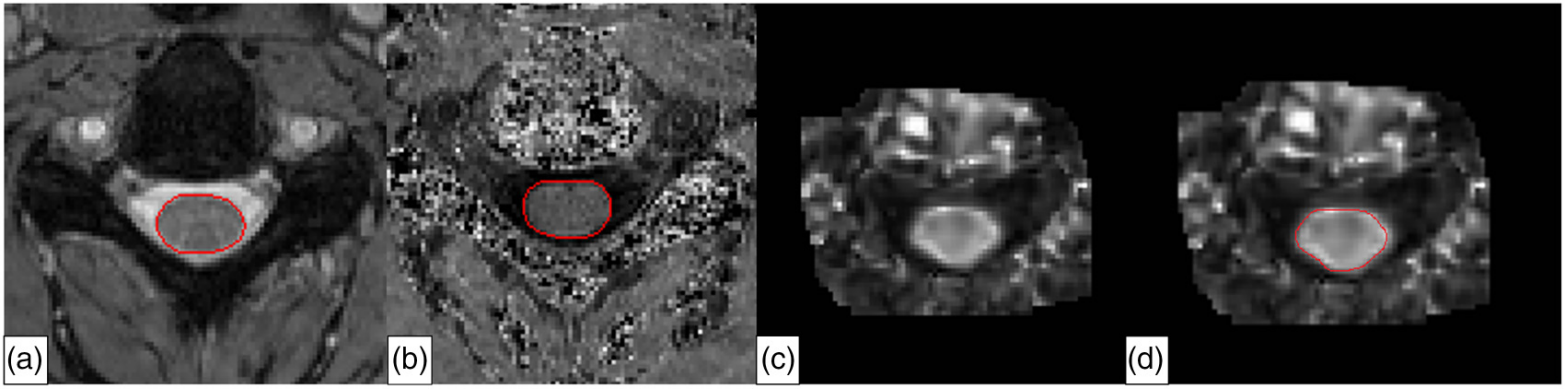
Quantitative spinal cord MRI analyses (a) Manual segmentation of spinal cord cross-sectional area on the MTon map; (b) Manual segmentation of region-of-interest (ROI) on magnetization-transfer ratio (MTR) map; (c) fractional anisotropy (FA) map; DManual segmentation of ROI on FA map.

*Spinal cord image analysis*. SC cross-sectional area (SC-CSA)—Automated segmentations of SC-CSA were performed on MT_on_ images using the Spinal Cord Toolbox (SCT) Software package.^32^ SC-CSA maps were reviewed for quality control by an experienced reader (ES) and excluded from the analysis if deemed to be of poor quality. Mean SC-CSA was calculated across the C3–C4 segment. (Figure 1).

*MTI analysis*—MT_on_ was registered to MT_off_ using a six degree-of-freedom, rigid-body process in FLIRT (Oxford Centre for Functional MRI of the Brain's Linear Imaging Registration Tool, Oxford, UK). (MT_off_ − MT_on_)/MT_off_ was used to calculate MTR (Figure 1). Due to technically inadequate images, one MTR map was excluded.

*DTI analysis*—Each diffusion-weighted image was registered to the initial b0 volume using a 6 degree-of-freedom, rigid-body registration in FLIRT using the Java Image Science Toolkit.^33^ The diffusion tensor and maps of DTI indices (fractional anisotropy [FA], mean diffusivity [MD], perpendicular diffusivity [λ_⊥_], and parallel diffusivity [λ_||_]), calculated from eigenvalues of the diffusion tensor, were produced.^17^ The b0 image was registered to the MT_off_ image using deformable transformation, and the extracted information applied to all diffusion-weighted images. Trilinear interpolation was used for deformable transformation, while resampling and rigid/affine transformation were performed using windowed sinc interpolation. Regions of interest (ROIs) were manually drawn on the FA maps across the C3–C4 segment (Figure 1) and transferred to the MD, λ_⊥_, and λ_||_ maps to obtain individual DTI indices. All segmentations were performed by a trained neurologist (DK) supervised by an MS physician experienced in spinal cord image analysis (ES). Visual inspection of all manual segmentations was performed, and slices were discarded if significant artifacts precluded accurate ROI delineation. This resulted in the exclusion of all DTI indices of one subject from the analysis.

*SC lesion count*. The presence of SC lesions was assessed on sagittal PSIR and STIR sequences for cervical SC and thoracic SC, respectively, and confirmed with axial MT sequences. SC lesion count was evaluated independently by two readers, an MS physician experienced in SC image analysis (ES) and an experienced neuroradiologist(SS). Two study subjects had confluent longitudinal lesions in the cervical SC which were considered as a single lesion in the final analysis.

### Data access

Upon request, research materials will be provided to qualified investigators.

### Statistical analysis

Statistical analyses were performed using STATA 15.0 (College Station, Texas). Spearman's correlation coefficient was used to assess correlations between variables. Multivariable linear regression analyses were performed to assess the impact of individual MRI measures on SD severity (dependent variable), while controlling for psychiatric symptomatology.

## Results

A total of 17 pwMS with self-reported SD were recruited. Study participants had a mean age of 46.9 years (SD10.3), 29.4% were female and 42% had a progressive disease course (either SPMS or PPMS). Mean disease duration was 15.05 (SD7.83) years, and median EDSS score was 3.0 (IQR 2–4). Eighty-three percent of participants were treated with disease-modifying treatments (DMTs). A past history of psychiatric comorbidity was common in our cohort, with 65% of participants having a psychiatric diagnosis noted in their medical record (anxiety, depression, substance abuse, or paranoid psychosis). Clinical characteristics and MRI measures of the study population are summarized in Table 1.

Table 2 summarizes the results of the questionnaires administered, which include measures of SD, sexual QoL, mood symptomatology, and fatigue. Eight percent (9/102) of the required questionnaires had incomplete responses and were not included in the analysis. All study participants had self-reported SD with a mean MSISQ-19 score of 40.73(SD13.71, range 22–65) and an SQoL score of 55.88 (SD17.10, range 31–96). Fifty-five percent (10/17) had moderate-to-severe depressive and anxious symptomatology based on the HADS and BDI-II scores.

**Table 2.** Questionnaire results.

Questionnaire	Questionnaire scores Mean (SD) Range	Test score reference
Multiple Sclerosis Intimacy and Sexuality Questionnaire-19 (MSISQ-19)	40.7 (13.7) 22–65	All items are scaled so that higher scores indicate a greater impact of MS on sexual function. Total score range: 19–95
Sexual Quality of Life Questionnaire (SQoL-M for men; SQoL-F for women)	55.9 (17.1) 31–96	All items are scaled so that higher scores indicate better QoL. To allow easy comparisons between genders, raw scores are standardized to a scale of 0–100
International Index of Erectile Function (IIEF) for men	14.1 (7.2) 6–26	1–10: Severe ED 11–16: Moderate dysfunction 17–21: Mild to moderate dysfunction 22–25: Mild dysfunction 26–30: No dysfunction
Female Sexual Function Index (FSFI) for women	17.7 (2) 16.5–20	A score <26.55 is classified as female sexual dysfunction
Beck Depression Inventory-II (BDI-II)	13.35 (6.7) 4–24	0–13 = minimal 14–19 = mild 20–28 = moderate 29–63 = severe
Hospital Anxiety and Depression Scale (HADS)	HADS-D 6.2 (3); 1–12 HADS-A 7.3 (4.7); 0–16	0–7 = normal 8–10 = borderline 11–21 = abnormal
Fatigue Impact Scale (FIS)	43.2 (16.7) 15–77	All items are scaled so that higher scores indicate a greater impact of fatigue on a person's activities. Total score range: 0–84

SD: standard deviation; QoL: quality of life; ED: erectile dysfunction.

On univariable analyses, there were no significant correlations between SD severity, as assessed by the SQoL, MSISQ-19, or IIEF/FSFI scores, and indicators of MS disease activity and severity such as annualized relapse rate (ARR), EDSS, disease duration, and disease subtype. Furthermore, the severity of SD did not correlate with any of the quantitative brain (GM, WM, lesion volumes, and BPF) or SC-MRI measures (SC-CSA, SC lesion count, MTR, and DTI indices). The only statistically significant correlations with SD severity, as assessed by the MSISQ-19, were found with anxiety as assessed by the HADS-A (ρ = 0.57, *p* = 0.02) and fatigue as assessed by FIS (*ρ* = 0.53, *p* = 0.04).

When SD severity (using the MSISQ-19 score) was assessed in multivariable analysis models with individual brain and SC-MRI measures included as the independent variables of interest, and mood/fatigue symptomatology (HADS, BDI-II, and FIS scores) as potentially confounding covariables; none of the brain and SC-MRI measures demonstrated independent statistically significant association with SD.

On the other hand, depression (BDI-II score) independently correlated with SD severity in models including brain atrophy measures (BPF and GM volumes, *p* = 0.03, *p* = 0.05, respectively). Anxiety (HADS-A score) showed an independent relationship with SD severity in several models which included SC measures: SC lesion count (*p* = 0.03), SC-CSA (*p* = 0.009), SC DTI indices (FA and λ_⊥_, *p* = 0.04, *p* = 0.05, respectively), and SC-MTR (*p* = 0.05). Additionally, fatigue (FIS score) showed independent correlations with SD severity in models including SC measures: DTI indices (MD and λ_||_,*p* = 0.05 for both models) and atrophy measures in the brain (BPF, GM, and WM volumes; *p* = 0.04, *p* = 0.01, *p* = 0.03, respectively).

Given that there is not an accepted gold standard questionnaire evaluating SD in MS, we included a number of questionnaires assessing SD that have different strengths (summarized in Supplemental Table 1). Further, we assessed for correlations between the different SD and fatigue questionnaires administered. There was no correlation found between the SD questionnaires; the MSISQ-19 and the SQoL. Considering the small study population, we did not perform a correlation analysis with the FSFI and the IIEF, which are gender-specific tools. We found a significant correlation between SD in pwMS as assessed by the MSISQ-19 score and fatigue as assessed by the FIS score; including all its subdomains (assessing physical, psychological, and cognitive fatigue) (*r* = −0.54, *p* = 0.04) and a trend toward a correlation between MSISQ-19 and mood symptoms (*r* = 0.46, *p* = 0.08). However, there was no correlation between SQoL and FIS.

## Discussion

SD is a highly prevalent symptom in pwMS but has received little attention in both clinical and scientific settings, with few prior studies having evaluated SD in pwMS in relation to quantitative MRI measures. In this pilot study of 17 pwMS with self-reported SD, we assessed the severity of SD in relation to quantitative MRI measures in the brain and SC, while controlling for clinical measures of relevance, including mood and fatigue symptomatology. Despite the fact that the SC is an important anatomic region mediating sexual function, we failed to find independent associations between the severity of SD and any of the quantitative SC or brain MRI measures we evaluated. The only variables that correlated with the severity of SD were the extent of fatigue, depression, and anxiety symptoms.

Prior studies that evaluated SD and various SC-MRI measures, including SC atrophy, SC lesion count, and location^16,21^ in pwMS similarly failed to find any correlations. When MRI measures in the brain were evaluated, there were no correlations with whole-brain atrophy. On the other hand, there have been inconsistent correlations demonstrated with specific brain regions (e.g., left insular region and pons) and brain lesion volumes with SD, which have not been replicated in subsequent studies.^16,22–25^

To our knowledge, DTI- and MTI-based SC measures, which are known to be more sensitive and specific to clinically relevant microstructural changes in the SC in MS in comparison to conventional MRI measures,^19,34^ have not previously been evaluated in relation to SD in pwMS. Despite using these measures, we did not find any significant association between SC-MRI measures and SD in pwMS. Furthermore, as has been described in prior studies, we did not identify any correlations between SD and various clinical measures of MS disease activity (disease duration, ARR, disease subtype, and disability level).^2,16,22^ Taken together, these findings suggest that SD in pwMS is multifactorial, and likely a result of a complex array of clinical, disease-related, social, and psychiatric factors, and not necessarily driven by disease severity or tissue damage in anatomic regions of relevance.

We included severity of mood and fatigue symptoms as potentially confounding variables, and identified clear univariable correlations between SD and fatigue, as well as SD and severity of mood symptoms which is in keeping with numerous prior studies. Prior work has identified depression and anxiety as important comorbidities influencing tertiary sexual dysfunction in pwMS.^13,35–37^ A systematic review exploring the association between SD and depression confirmed a bidirectional association that can often form positive pathological feedback cycles,^38^ which underscores the degree these symptoms are interrelated. Another study demonstrated that the most frequent symptoms of SD in pwMS are lack of sexual desire and arousal and the inability to reach orgasm,^5^ which are often symptoms linked to psychiatric comorbidities rather than physical disability.^5,13,35,37^ When we evaluated multivariable models including individual MRI measures of interest and measures of fatigue, anxiety, and depression as confounding covariates, we found that while SD did not correlate with any of the MRI measures, it did retain independent correlations with the severity of neuropsychiatric symptoms. The fact that measures of mood and fatigue symptoms independently correlated with SD above and beyond any advanced quantitative brain or SC-MRI measures further reinforces the important relationship between these complex symptoms and the importance of recognizing and treating mental health comorbidities in pwMS. As this was a pilot cross-sectional study, we were unable to evaluate causality with respect to the relationship between mood symptoms and SD—however, this would be an important area for future study.

Our findings have implications for clinical approaches to treating SD in pwMS. They highlight the need to consider mood and fatigue symptoms when attempting to address symptoms related to sexual health. However, given that SD is a complex entity that is related to multiple factors, which include but are not limited to MS disease processes as well as mood symptoms and fatigue, clinicians should approach this topic with sensitivity and discuss the complexity and possible links with mood with patients in a holistic manner, with the goal of using multiple approaches to improve symptoms. Despite the high prevalence of SD in pwMS, treatment options, particularly for tertiary SD, are limited. A recent systematic review showed beneficial effects of psychobehavioral treatment approaches for sexual impairment and sexual satisfaction in pwMS; though the quality of evidence was weak due to the small sample size of studies included.^39^

We included numerous questionnaires evaluating SD as there is a lack of clarity regarding the best instrument to use to evaluate SD in pwMS. We found there was no correlation between MSISQ-19 and SQoL. Moreover, there was only a correlation between fatigue and MSISQ-19, which was further highlighted in our multivariable models. Taken together, these findings suggest that MSISQ-19 may be a better instrument to evaluate SD in MS and highlight the importance of using disease-specific tools to evaluate complex symptoms such as SD.

Our study has several limitations. First, the small sample size makes it difficult to draw definitive conclusions. The lack of a correlation that we observed with specific MRI measures may be related to the lack of adequate power in the study. Despite this, the fact that we were still able to identify correlations with mood and fatigue symptom severity underscores the strength of the observed association. Second, as our study participants were recruited based on self-reported SD and self-referral, our study population is not reflective of a typical chronic MS population and therefore likely lacks external validity. This is evident by the fact that the majority of participants were male, which is atypical for a chronic MS cohort. The male predominance in our cohort is likely related to sex differences in perceiving and discussing SD with health care providers, as well as a willingness to self-refer. Psychological aspects affecting sexual function and neuroimaging findings likely vary between sexes as well; thus, our findings may not be widely applicable to female patients. Our small sample size did not allow us to evaluate gender effects on SD in pwMS, which is of interest, and should be evaluated in future studies. Third, we did not include pwMS that do not suffer from SD as study controls and were therefore not able to evaluate if SD is categorically associated with quantitative MRI measures. Furthermore, we did not have a healthy control population for comparative purposes, which limit our ability to draw definitive conclusions. Further studies are needed to better characterize SD and its subdomains (which include well-defined symptoms) in a wider range of pwMS, using additional relevant quantitative MRI measures, as there may be differences across genders, disease subtypes, disease severities, comorbidities, and ethnicities.

In conclusion, we found that the severity of SD in pwMS does not correlate and is independent of MS-related tissue changes in the brain and SC as evaluated by quantitative brain and SC-MRI measures. The only variable that consistently retained a relationship with SD was psychiatric symptoms, suggesting that there is a relationship between SD and psychiatric well-being. These findings highlight the complex, multifactorial nature of SD in pwMS and underscore the importance of taking into account psychobehavioral symptomatology when devising effective treatment approaches for SD in pwMS, which is an unmet need in clinical practice.

## Supplemental Material

sj-docx-1-mso-10.1177_20552173221132170 - Supplemental material for Quantitative spinal cord MRI and sexual dysfunction in multiple sclerosis Click here for additional data file. Supplemental material, sj-docx-1-mso-10.1177_20552173221132170 for Quantitative spinal cord MRI and sexual dysfunction in multiple sclerosis by Estelle Seyman, David Kim, Aditya Bharatha, Courtney Casserly, Kristen Krysko, Roy-Hewitson Chantal, Paula Alcaide-Leon, Suradech Suthiphosuwan and Jiwon Oh in Multiple Sclerosis Journal – Experimental, Translational and Clinical

sj-doc-2-mso-10.1177_20552173221132170 - Supplemental material for Quantitative spinal cord MRI and sexual dysfunction in multiple sclerosis Click here for additional data file. Supplemental material, sj-doc-2-mso-10.1177_20552173221132170 for Quantitative spinal cord MRI and sexual dysfunction in multiple sclerosis by Estelle Seyman, David Kim, Aditya Bharatha, Courtney Casserly, Kristen Krysko, Roy-Hewitson Chantal, Paula Alcaide-Leon, Suradech Suthiphosuwan and Jiwon Oh in Multiple Sclerosis Journal – Experimental, Translational and Clinical
