# Supplementary material for: Quantitative spinal cord MRI and sexual dysfunction in multiple sclerosis
Source: Mult Scler J Exp Transl Clin. 2022 Oct 20;8(4):20552173221132170. doi: 10.1177/20552173221132170 (PMC9585573; doi:10.1177/20552173221132170)
Supplement: sj-docx-1-mso-10.1177_20552173221132170 - Supplemental material for Quantitative spinal cord MRI and sexual dysfunction in multiple sclerosis [file sj-docx-1-mso-10.1177_20552173221132170.docx]

**Supplemental Table 1 – Summary of Questionnaires**

| Questionnaire | Description and validation | Test score reference |
| --- | --- | --- |
| Multiple Sclerosis Intimacy and  Sexuality Questionnaire-19 (MSISQ-19) | A disease-specific questionnaire evaluating SD that measures the frequency of various sexual symptoms over the preceding six months. This is the most widely used instrument to investigate sexual function in pwMS and has demonstrated reliability as well as construct, concurrent, and criterion validity in pwMS (1) | All items are scaled so that higher scores indicate a greater impact of MS on sexual function. The total score ranges 19-95. |
| Sexual Quality of Life Questionnaire; (SQoL-M for men; SQoL-F for women) | Valid instruments for assessing the impact of SD on quality of life measures and sexual QoL in men and women. Both instruments have demonstrated good psychometric properties in patients suffering from neurological conditions (2) and showed excellent discriminant and convergent validity for assessing SD in the general population (3-5) . | All items are scaled so that higher scores indicate better QoL. To allow easy comparisons between genders, raw scores are standardized to a scale of 0 to 100 |
| International Index of Erectile Function (IIEF) for men | Quantifies erectile dysfunction severity in health and disease, and has been validated for use in men with MS (6, 7) | 1-10: Severe erectile dysfunction  11-16: Moderate erectile dysfunction  17-21: Mild to moderate erectile dysfunction  22-25: Mild erectile dysfunction  26-30: No dysfunction |
| Female Sexual Function Index (FSFI) for women | Evaluates key dimensions of sexual function in women (8), was used and validated in women with MS (9) | A score < 26.55 is classified as female SD. |
| Beck Depression Inventory–II  (BDI-II) | One of the most widely used instruments to measure depression severity and has been validated for use in pwMS (10, 11) | 0-13= Minimal  14-19= Mild  20-28= Moderate  29-63 = Severe |
| Hospital Anxiety and Depression Scale (HADS) | Measures symptoms of anxiety and depression with good reliability and discriminant validity in pwMS. (12) | 0-7 = Normal  8-10 = Borderline  11-21 = Abnormal |
| Fatigue Impact  Scale (FIS) | Measures the severity and impact of fatigue and has been validated for use in pwMS with good reliability and precision (13-15). | All items are scaled so that higher scores indicate a greater impact of fatigue on a person’s activities. The total score can range from 0 to 84 |

Legend: SD = sexual dysfunction, MS = multiple sclerosis, pwMS = people with MS, QoL= Quality of life.

**References**:

1. Foley FW, Zemon V, Campagnolo D, Marrie RA, Cutter G, Tyry T, et al. The Multiple Sclerosis Intimacy and Sexuality Questionnaire -- re-validation and development of a 15-item version with a large US sample. Mult Scler. 2013;19(9):1197-203.

2. t Hoen LA, Groen J, Scheepe JR, Reuvers S, Diaz DC, Fernandez BP, et al. A Quality Assessment of Patient-Reported Outcome Measures for Sexual Function in Neurologic Patients Using the Consensus-based Standards for the Selection of Health Measurement Instruments Checklist: A Systematic Review. Eur Urol Focus. 2017;3(4-5):444-56.

3. Abraham L, Symonds T, Morris MF. Psychometric validation of a sexual quality of life questionnaire for use in men with premature ejaculation or erectile dysfunction. J Sex Med. 2008;5(3):595-601.

4. Symonds T, Boolell M, Quirk F. Development of a questionnaire on sexual quality of life in women. J Sex Marital Ther. 2005;31(5):385-97.

5. Hatzichristou D, Kirana PS, Banner L, Althof SE, Lonnee-Hoffmann RA, Dennerstein L, et al. Diagnosing Sexual Dysfunction in Men and Women: Sexual History Taking and the Role of Symptom Scales and Questionnaires. J Sex Med. 2016;13(8):1166-82.

6. Rosen RC, Riley A, Wagner G, Osterloh IH, Kirkpatrick J, Mishra A. The international index of erectile function (IIEF): a multidimensional scale for assessment of erectile dysfunction. Urology. 1997;49(6):822-30.

7. Calabro RS, De Luca R, Conti-Nibali V, Reitano S, Leo A, Bramanti P. Sexual dysfunction in male patients with multiple sclerosis: a need for counseling! Int J Neurosci. 2014;124(8):547-57.

8. Rosen R, Brown C, Heiman J, Leiblum S, Meston C, Shabsigh R, et al. The Female Sexual Function Index (FSFI): a multidimensional self-report instrument for the assessment of female sexual function. J Sex Marital Ther. 2000;26(2):191-208.

9. Gava G, Visconti M, Salvi F, Bartolomei I, Seracchioli R, Meriggiola MC. Prevalence and Psychopathological Determinants of Sexual Dysfunction and Related Distress in Women With and Without Multiple Sclerosis. J Sex Med. 2019;16(6):833-42.

10. Watson TM, Ford E, Worthington E, Lincoln NB. Validation of mood measures for people with multiple sclerosis. Int J MS Care. 2014;16(2):105-9.

11. Avasarala JR, Cross AH, Trinkaus K. Comparative assessment of Yale Single Question and Beck Depression Inventory Scale in screening for depression in multiple sclerosis. Mult Scler. 2003;9(3):307-10.

12. Honarmand K, Feinstein A. Validation of the Hospital Anxiety and Depression Scale for use with multiple sclerosis patients. Mult Scler. 2009;15(12):1518-24.

13. Fisk JD, Ritvo PG, Ross L, Haase DA, Marrie TJ, Schlech WF. Measuring the functional impact of fatigue: initial validation of the fatigue impact scale. Clin Infect Dis. 1994;18 Suppl 1:S79-83.

14. Learmonth YC, Dlugonski D, Pilutti LA, Sandroff BM, Klaren R, Motl RW. Psychometric properties of the Fatigue Severity Scale and the Modified Fatigue Impact Scale. J Neurol Sci. 2013;331(1-2):102-7.

15. Flachenecker P, Kumpfel T, Kallmann B, Gottschalk M, Grauer O, Rieckmann P, et al. Fatigue in multiple sclerosis: a comparison of different rating scales and correlation to clinical parameters. Mult Scler. 2002;8(6):523-6.
