# Supplementary material for: Quantitative spinal cord MRI and sexual dysfunction in multiple sclerosis
Source: Mult Scler J Exp Transl Clin. 2022 Oct 20;8(4):20552173221132170. doi: 10.1177/20552173221132170 (PMC9585573; doi:10.1177/20552173221132170)
Supplement: sj-doc-2-mso-10.1177_20552173221132170 - Supplemental material for Quantitative spinal cord MRI and sexual dysfunction in multiple sclerosis [file sj-doc-2-mso-10.1177_20552173221132170.doc]

**MS Journal Appendix for MRI methodology**

| Hardware | |
| --- | --- |
| Field strength | 3T |
| Manufacturer | Siemens |
| Model | Skyra |
| Coil type  (e.g. head, surface) | 20-channel head-neck coil and a 16-channel spine-array coil. |
| Number of coil channels | 20-channel head-neck coil and a 16-channel spine-array coil. |

| Acquisition sequence | | |
| --- | --- | --- |
| Type  (e.g. FLAIR, DIR, DTI, fMRI) | Brain sequences: T1-weighted magnetization-prepared rapid acquisition gradient echo (T1-MPRAGE) : TR/TE/Flip angle = 1,900 ms/2.52 ms/9°, parallel acceleration technique mode=GRAPPA, acceleration factor=2, slice thickness=1 mm, field-of-view (FOV) = 250 mm, in- plane resolution = 1×1 mm2, and number of slices = 176 and T2-weighted fluid-attenuated inversion recovery (FLAIR): TR/TE = 4,800 ms/353 ms, TI = 1,800 ms, parallel acceleration technique mode = GRAPPA, acceleration factor = 2, slice thick- ness = 1 mm, FOV = 256 mm, in-plane resolution = 1×1 mm2, number of slices=176.  Spinal Cord Sequences: Sagittal 2D T1-weighted phase-sensitive inversion recovery (PSIR) of the cervical spine (24): FOV = 220 mm; in-plane resolution = 0.7 × 0.7 mm; slice thickness = 3 mm; TR/TE = 2,400/9.4 ms; TI = 400 ms; averages = 2; parallel acceleration technique mode = GRAPPA; and acceleration factor = 2.  Sagittal 2D T1- Short-TI -Inversion Recovery (STIR) of the thoracic spine: FOV = 220 mm; in-plane resolution = 0.48 × 0.48 mm; slice thickness = 3 mm; TR/TE = 4,000/50 ms; TI = 200 ms; averages = 1; parallel acceleration technique mode = GRAPPA; and acceleration factor = 2.  MTI data: MT-weighted images (MTon): 3D T2*-weighted, gradient-echo sequence with an MT prepulse (1.5-kHz off-resonance sincgauss–shaped radiofrequency saturation pulse), flip angle/ TR/TE = 9°/47 ms/11.2 ms, which yielded 3-mm axial slices (20 contiguous) spanning C3-C4 with FOV = 224 mm, and a nominal in-plane resolution of 0.6 × 0.6 mm2. MToff images used the same parameters but excluded the MT prepulse.  DTI data: cardiac-gated, axial fat-suppressed, high-resolution diffusion-weighted imaging with readout-segmented echo- planar imaging, parallel imaging, and a 2-dimensional navigator-based reacquisition (RESOLVE) was obtained across C3-C4 in 12 noncoplanar gradient directions. Flip angle/TR/TE = 180°/220 ms/53 ms, b = 500 seconds/mm2, trigger delay = 0 ms, acquisition window = 420 ms, parallel acceleration technique mode = GRAPPA, acceleration factor = 2, slice thickness = 3 mm, FOV = 150 mm, in-plane resolution = 1.5×1.5 mm2, and slice number = 20. | |
| Acquisition time | As above | |
| Orientation | As above | |
| Alignment  (e.g. anterior commissure/poster commissure line) | As above | |
| Voxel size | As above | |
| TR | As above | |
| TE | As above | |
| TI | As above | |
| Flip angle | As above | |
| NEX | As above | |
| Field of view | As above | |
| Matrix size | As above | |
| Parallel imaging | Yes – for DTI sequence | No |
| If used, parallel imaging method:  (e.g. SENSE, GRAPPA) | GRAPPA – as above | |
| Cardiac gating | Yes – for DTI sequence | No |
| If used, cardiac gating method:  (e.g. PPU or ECG) | PPU | |
| Contrast enhancement |  | No |
| If used, provide name of contrast agent, dose and timing of scan post-contrast administration | n/a | |
| Other parameters: | As above | |

| Image analysis methods and outputs | |
| --- | --- |
| ***Lesions*** | |
| Type  (e.g. Gd-enhancing, T2-hyperintense, T1-hypointense) | T2-hyperintense and T1-hypointense in spinal cord |
| Analysis method | Visual inspection (using PSIR sequence for cervical spinal cord lesions and STIR for thoracic spinal cord lesions) |
| Analysis software | n/a |
| Output measure  (e.g. count or volume [ml]) | Count |
| ***Tissue volumes*** | |
| Type  (e.g. whole brain, grey matter, white matter, spinal cord) | Gray matter volume, white matter volume, CSF volume, brain parenchymal fraction, spinal cord cross-sectional area (SC-CSA) |
| Analysis method | Substructure segmentation |
| Analysis software | Lesion Segmentation Toolbox for SPM8 (Department of Imaging Neuroscience, London, UK) on MATLAB software.  Spinal cord toolbox for SC-CSA – calculated across C3-C4 |
| Output measure  (e.g. absolute tissue volume in ml, tissue volume as a fraction of intracranial volume, percentage change in tissue volumes) | BPF: brain parenchymal volume(GM + WM) divided by total intracranial volume (GM + WM + CSF); normalize gray matter volume (GM / intracranial volume), spinal cord cross-sectional area |
| ***Tissue measures (e.g. MTR, DTI, T1-RT, T2-RT, T2*, T2’, ^1^H-MRS, perfusion, Na)*** | |
| Type  (e.g. whole brain, grey matter, white matter, spinal cord, normal-appearing grey matter or white matter) | Spinal cord DTI and MTR |
| Analysis method | Each diffusion-weighted image was registered to the initial b0 volume using a 6 degree-of-freedom, rigid-body registration in FLIRT using the Java Image Science Toolkit . The diffusion tensor and maps of DTI indices (fractional anisotropy[FA], mean diffusivity[MD], perpendicular diffusivity[λ_⊥_], and parallel diffusivity[λ||]), calculated from eigen values of the diffusion tensor, were produced . The b0 image was registered to the MT_off_ image using deformable transformation, and the extracted information applied to all diffusion-weighted images. Trilinear interpolation was used for deformable transformation while resampling, and rigid/affine transformation was performed using windowed sinc interpolation.  MT_on_ was registered to MT_off_ using a 6 degree-of-freedom, rigid-body process in FLIRT (Oxford Centre for Functional MRI of the Brain’s Linear Imaging Registration Tool, Oxford, UK). (MT_off_ − MT_on_)/MT_off_ was used to calculate MTR.  Regions of interest(ROI) were manually drawn on the FA maps across the C3-C4 segment (Figure 1) and transferred to the MD, λ_⊥_, and λ|| and MTR maps to obtain individual DTI indices and MTR. |
| Analysis software | Java Image Science Toolkit |
| Output measure | MTR, DTI indices (FA, MD, λ_⊥_, λ||]) |
| ***Other MRI measures (e.g. functional MRI)*** | |
| Type  (e.g. whole brain, grey matter, white matter, spinal cord, normal-appearing grey matter or white matter) |  |
| Analysis method |  |
| Analysis software |  |
| Output measure |  |

**Other analysis details:**
